# Innovative silver-salicylic acid nanoparticle coatings based on CMC and chitosan for mango (*Mangifera indica* L. cv. “Fajri Klan”) fruit preservation

**DOI:** 10.1038/s41598-026-52586-5

**Published:** 2026-05-19

**Authors:** Ibrahim Hmmam, Abdou Abdallatif, Bahaaaldin Mohamed Mamdouh, Mohamed A. Ali

**Affiliations:** 1 Pomology Department, Faculty of Agriculture, Cairo University, Giza, 12613 Egypt; 2 Biochemistry Department, Faculty of Agriculture, Cairo University, Giza, 12613 Egypt

**Keywords:** AgNPs, Chitosan, CMC, Cold storage, Edible coating, *Mangifera indica*, Nanoparticles characterization, Shelf life, Biochemistry, Biotechnology, Chemistry, Materials science, Microbiology, Nanoscience and technology, Plant sciences

## Abstract

This research explores synthesis of silver-salicylic acid nanoparticles (AgNPs-SA) and their integration with carboxymethyl cellulose (CMC) to form CMC-AgNPs-SA and with chitosan to form Ch-AgNPs-SA. The effects of these composite materials were evaluated in terms of microbiological activity, storage performance, physicochemical attributes, and shelf-life extension of ‘Fajri Klan’ mangoes fruits at 13 °C. The synthesized AgNPs-SA was confirmed to be spherical, with sizes between 20.9 and 33.4 nm and a zeta potential of -20 mV. Additionally, CMC-AgNPs-SA, and Ch-AgNPs-SA demonstrated comparable antibacterial efficacy against *Escherichia coli* and *Staphylococcus aureus*. Over the storage period, cold-stored mangoes exhibited gradual increased in weight loss, respiration rate, and higher levels of total soluble solids (TSS), total sugars, and carotenoids. However, these changes were more pronounced in the uncoated samples (control). The application of CMC-AgNPs-SA and Ch-AgNPs-SA helped maintain fruit quality, suppressed decay, and extended shelf life. Although firmness and titratable acidity (TA) naturally declined with time, the coated mangoes retained these qualities better than the control group. Based on these findings, CMC-AgNPs-SA and Ch-AgNPs-SA show strong potential as effective coatings for preserving fruit quality and prolonging the storage duration of ‘Fajri Klan’ mangoes.

## Introduction

Mango (*Mangifera indica* L. Family; Anacardiaceae) is a climacteric fruit which is normally harvested at physiological maturity stage^1^, and then undergo various biochemical changes to achieve the desired taste, flavor and texture^2^. During fruit ripening, ethylene triggers number of biochemical reactions results in changes in flesh and skin color, texture change and increase fruit juiciness, conversion of starch into soluble sugars, reduced acidity and accumulation of aroma components^3^. Mango pulp contains various types of biologically active compounds such as dietary fiber, minerals, organic acids, vitamins, and polyphenols, that contributes towards the fruit distinctive flavor and nutritional value, which make it a favorite choice for consumers^4,5^. However, mango fruit characterized with a short shelf life, susceptibility to mechanical damage, chilling injury and storage pathogens which resulting in decline of fruit quality, marketing value and high postharvest loss^1,4,6,7^. Maintenance of fruit quality through marketing chain is depending on development of suitable technology at different stages of harvesting, handling and storage^7^. Maintenance of fruit quality and minimizing decay incidence can be achieved by several strategies including low temperature storage, coating, and application of controlled and modified atmosphere storage^8,9^. Among these techniques edible coatings offer an attractive environment-friendly approach to extend potential storage shelf life of mango fruit through retard water loss, gas exchange, delay ripening related biochemical reactions and maintain sensory attributes, inhibiting the growth of microorganisms and improve fruit appearance^1,4,7,10^. Edible coatings with natural materials i.e. cellulose derivatives, biopolymers, proteins, lipids, chitin and pectin considered as a safe, stable, biodegradable and environment-friendly preservation material^11,12^. Chitosan a naturally polysaccharide derived from deacetylated of chitin^13^, exhibits excellent gas–barrier properties and anti-microbial activity^14^. Carboxymethylcellulose is cellulose derivative composed of linear anionic chains of glucopyranosyl units, provided stable nontoxic and biodegradable coating substances^15^. Edible coatings based on CMC have been effective in preserving the postharvest quality of avocado, citrus, guava, mango and plum^12,16–18^. Edible coating can be used as a carrier of functional ingredients, such as antimicrobial, antioxidant, flavor, and colorants substances^19^. The application of nanotechnology is a new approach for extending storage life, reduces postharvest loss and maintains quality of fresh fruit^16,20^. The unique nanoparticles properties provide high physical and chemical activity in addition to antimicrobial properties^21,22^. Recently, the use of coating materials loaded with nanoparticles represented an innovative fruit preservation strategy that guarantees minimal exposure of nanomaterials to the stored fruits^20,23,24^. Coating materials mixed with silver nanoparticles can be applied to control microorganisms’ activity that cause postharvest pathological decay and maintain fruit quality during cold storage and increase shelf life^16,23,25,26^. Therefore, the current work aims to study the impact of coating with chitosan and Carboxymethyl cellulose loaded with AgNPs-SA on storage behavior and quality parameters of ‘Fajri Klan’ mango cultivar.

## Materials and methods

### Preparation of CMC and chitosan based AgNPs-SA coatings

#### Preparation of coating base

A 1.0% chitosan solutions (w/v) was formulated following the procedure described by **Abdipour** et al.^27^. Specifically, 10.0 g of low MW chitosan (degree of deacetylation exceeding 80%; Oxford Lab Fine Chem, India) was dissolved in 1,000 mL of deionized water with the addition of 20 mL of glacial acetic acid (Merck, Germany) by continuous agitation for 2 h, followed by the subsequent pH correction to 4.8 using a 0.1 M NaOH (Merck, Germany). Concurrently, a separate 2% (w/v) carboxymethyl cellulose (CMC) solution was prepared by dispersing 20 g of CMC powder (LOBA Chemie, India) into a 1,000 mL aqueous-ethanol solvent system with a 3:1 volume ratio at 80 °C for 1 h^28^.

#### Preparation of silver nanoparticles loaded salicylic acid (AgNPs-SA)

Silver nanoparticles incorporating salicylic acid (AgNPs-SA) was synthesized via a phenolic acid reduction approch^29^, wherein salicylic acid (SA) served a dual role as both a reducing and stabilizing agents. The synthesis commenced with the preparation of a 4 mM SA solution in 18 mL of deionized water. The pH of this solution was adjusted to 10.5 by the addition of 0.1 M NaOH. This alkaline SA solution was subsequently introduced in a dropwise manner into 100 mL of a 2 mM silver nitrate (AgNO₃) solution. The combined mixture was then heated to 100 °C in boiling water bath for approximately 2 h until the color turned from colorless to a characteristic yellowish-brown color which, indicating the metal ion reduction and formation of AgNPs-SA.

#### Preparation of CMC and chitosan based AgNPs-SA coatings

The previously synthesized CMC and chitosan base solutions were independently combined with the AgNPs-SA suspension in a 1:1 volumetric ratio^23^. This procedure yielded two distinct functional coatings: a carboxymethyl cellulose-based silver nanoparticle formulation (CMC-AgNPs-SA) and a chitosan-based silver nanoparticle formulation (Ch-AgNPs-SA).

### Characterization of silver nanoparticles

#### Particle size and zeta potential

A Zetasizer^®^ 3000 particulate size characterization analyzer (Malvern Instruments, UK, England) in the Faculty of Pharmacy’s central lab was employed to determine zeta potential and mean particle size of the nanoparticulate samples via photon correlation spectroscopy (PCS) and laser Doppler anemometry (Malvern Instruments, UK, England), correspondingly. In brief, the size was evaluated three times at 25 °C and a scattering angle of 90°. Each measurement was taken for 3 min. The final hydrodynamic diameter was derived from a cumulative assessment of the acquired data. The zeta potential was determined using an automated aqueous dip cell.

### Morphological characteristics by transmission electron microscopy (TEM)

The morphological characteristics of the synthesized nanoparticles were examined using transmission electron microscopy (TEM) at the Cairo University Research Park (CURP). For sample preparation, a 2 µL aliquot of an aqueous nanoparticle suspension was deposited onto a carbon-coated copper grid. Imaging was conducted with JEOL JEM-1400 electron microscope, JEOL Ltd., Akishima, Japan operating at an accelerating voltage ranging from 40 to 120 kV. This analysis was performed.

### Scanning electron microscopy (SEM) and energy-dispersive X-ray spectroscopy (EDX)

The fabricated AgNPs-SA were subjected to a gold-coating process using an S150A sputter coating unit (Edwards, England). Nanoparticle surface structure and texture were subsequently examined via field emission scanning electron microscopy (FE-SEM) using a Quanta FEG 250 apparatus (Holland) at the Electron Microscopy Unit of Egypt’s National Research Centre. A drop of the suspended AgNPs-SA was placed on a cleaned glass slide to create the SEM samples, which were then allowed to dry at room temperature until all of the water had evaporated. The sputter coater was used for three minutes to gold plate the glass slide. Additionally, the elemental composition of the coated samples was evaluated by energy-dispersive X-ray spectroscopy (EDS), which was conducted with the microscope’s built-in software suite (TEAM).

### Fourier transform infrared spectroscopy (FTIR)

The degree of material association during AgNPs-SA synthesis was analyzed at the Central Laboratory of the National Research Center using FTIR (VERTEX 80v, BRUKER, Germany). Data acquisition was conducted with a spectral resolution of 4 cm⁻¹ across a wavenumber range from 4000 to 400 cm⁻¹.

### Antbactirial activities of prepared NPs

The antimicrobial activity of the prepared NPs was assessed against Gram- negative *Escherichia coli* O157:H7 (wild type strain 93,111) and Gram- positive *Staphylococcus aureus* (ATCC 25923) sourced from the Cairo University Research Park (CURP), Egypt, using the agar well diffusion method^30^. One mL of bacterial culture was inoculated into a sterile Petri dish filled with Mueller–Hinton agar medium. After the culture medium was completely solidified, three wells with a 10 mm diameter were created. A total of 100 µL of 1% AgNPs-SA, CMC-AgNPs-SA, and Ch-AgNPs-SA formulations (w/v) was introduced into the wells, and the plates were incubated at 37 °C for 24–48 h, polymyxin at the same volume and concentration being used as the control. The antibacterial properties of the samples were quantified by measuring the diameter of the inhibition zone in millimeters (mm).

### Fruits material

Mature ‘Fajri Klan’ mango fruits were collected at the physiological maturity stage from a commercial orchard under standard horticulture management in El-Behara Governorate, Egypt. Upon harvest, fruits were promptly sent to the postharvest laboratory of the Pomology Department. Selection criteria involved choosing uniform, ripe, and firm specimens that were free from visible mechanical injury or pathological symptoms for the subsequent coating applications. Prior to treatment, the mango fruits were rinsed under flowing tap water and air-dried at ambient temperature.

### Coating application and storage conditions

The selected mango fruits were divided into four distinct treatment groups, with each containing 60 fruits. Groups were assigned as follows; three groups were dipped for 60 s in solutions of AgNPs-SA, CMC-AgNPs-SA, or Ch-AgNPs-SA, while the fourth group served as an uncoated control. Following the immersion treatment, all fruits were allowed to dry under ambient conditions, the treated fruits were subsequently packaged in cardboard boxes with ten fruits per box, and placed into cold storage at 13 °C and 85–90% relative humidity. Throughout the 28-day storage duration, key physicochemical attributes of the fruit from both coated and control groups were assessed at weekly intervals.

### Fruit quality attributes

#### Respiration rate

From each treatment group, three mango fruits of predefined mass and dimensions were enclosed in separate 2-liter airtight containers and stored at 13 °C for 24 h. Following this incubation period, headspace gas samples were collected for carbon dioxide determination using a portable YesAir gas analyzer (IAQ monitor, Delta, BC, Canada), which was fitted with integrated O_2_ and CO_2_ sensors. The fruit respiration rate was subsequently calculated using the method described by Pristijono et al.^31^ and expressed in units of nmol CO₂ kg⁻¹ s⁻¹.

#### Weight loss

Fruit weight loss was monitored regularly at specific time intervals (7 d). Fruit samples (*n* = 10) of each coating treatment were weighed at the beginning of storage time (W1) and at each sampling date (W2) using an electronic balance, weight loss (%) was calculated according to **Ali et al.**^32^ by the Eq. (1)

1 $${\text{Weight loss }}\left( \% \right){\text{ }} = {\text{ }}\left( {{\mathrm{W1}} - {\mathrm{W2}}} \right)/{\mathrm{W1}}\times 100$$

#### Fruit pulp firmness

Fruit pulp firmness (*n* = 3) was determined according to **Liu et al.**^33^ using firmness tester (Lutron FR-5120, Electronic Enterprise, Taipei, Taiwan) equipped with a 5 mm stainless steel round-bottomed probe. The firmness was recorded at two opposite points of fruit surface in the equatorial zone.

#### Total soluble solids (TSS), titratable acidity (TA), and TSS/TA ratio

Total soluble solids (TSS) were quantified by placing several drops of extracted fruit juice onto the prism of a digital refractometer (Pocket refractometer PAL-1, Atago, Japan) with results presented as a percentage. For titratable acidity (TA), 10 mL of a diluted mango juice preparation (1 mL juice + 9 mL distilled water) was titrated with 0.1 N sodium hydroxide, using phenolphthalein as an indicator until the endpoint (a persistent faint pink color) was reached. TA was calculated as a percentage of citric acid relative to the fresh pulp weight, following a standard method^34^. The TSS/TA ratio for each treatment was determined by dividing the TSS value by its respective titratable acidity value.

#### Total sugars content

Total sugars content was assessed according to the phenol-sulfuric acid method^23^. Briefly, total sugars were obtained from 0.25 g fruit pulp by extraction with 20 mL of 70% ethanol. A 1 mL aliquot of this ethanolic extract was then combined with 1 mL of 5% phenol and 5 mL of concentrated (98%) H_2_SO_4_. This mixture was left undisturbed for 1 h to allow color development. The resultant absorbance of the chromogenic solution was measured at 490 nm on a UV-Vis spectrophotometer (JENWAY 6300, UK). Quantification was achieved by reference to a calibration curve constructed using known concentrations of glucose standard, and results were reported as mg glucose equivalent g of fresh weight.

#### Total carotenoids

Carotenoid pigments were isolated from 0.25 g of fruit pulp by mixing the sample with 20 mL of an 80% acetone solution. The optical density of the resulting extract was recorded at wavelengths of 480 and 510 nm using a UV-visible spectrophotometer (JENWAY, Model 6300, UK). The total carotenoids content was calculated according to **Jensen**^35^ by using the following equation:

2 $${\text{Total Carotenoids }}\left( {\mu {\text{g g}}^{{ - {\mathrm{1}}}} } \right){\text{ }} = {\text{ 7}}.{\text{6 }}\left( {OD{\mathrm{48}}0} \right){\text{ }}{-}{\text{ 1}}.{\text{49 }}\left( {OD{\mathrm{51}}0} \right)\times\left( {V/{\mathrm{1}}000} \right)\times\left( W \right)$$

where OD = optical density, V = final volume of 80% acetone, W = sample weight.

#### Total phenol (TPC)

TPC was assessed using Folin–Ciocalteu assay^36^. Extraction of TPC was performed by homogenizing 0.5 g of mango fruit pulp in 20 ml methanol (Chem-Lab., Zedelgem, Belgium), 1 ml of Folin-Ciocalteu’s reagent (LOBA Chemie PVT. Ltd., Mumbai, India) was mixed with 1 ml of methanolic extract for 6 min. Then, 4 mL of 1 M sodium carbonate (LOBA Chemie PVT. Ltd., Mumbai, India) and 3 mL of water were added to the solution and incubated in darkness at room temperature for 90 min. The absorbance of the assay mixture was measured at 760 nm (JENWAY, Model 6300, Staffordshire, UK). Gallic acid was used to generate a calibration curve, and TPC was reported as mg of gallic acid equivalents per g of fresh pulp weight.

### DPPH free radical scavenging assay

Antioxidant capacity was assessed using the DPPH free radical scavenging assay as outlined by Rahman et al.^37^. Methanolic fruit extracts, prepared by homogenizing 0.5 g of mango pulp in 20 mL of methanol, were used for the analysis. For the assay, 1.6 mL of this extract was combined with 2.4 mL of a 0.004% methanolic solution of 1,1-diphenyl-2-picrylhydrazyl (DPPH; Sigma-Aldrich, Germany). The resultant mixture was thoroughly agitated using a vortex mixer and then kept in the dark at ambient temperature for 30 min. Following incubation, the absorbance of each sample was measured at a wavelength of 517 nm with a spectrophotometer. The DPPH radical scavenging activity was calculated by the following equation:

3 $${\text{DPPH radical scavenging activity }} = {\text{ }}\left( {{\mathrm{A}}_{{{\mathrm{517}}}}\, {\text{control }}{-}{\text{ A}}_{{{\mathrm{517}}}}\, {\mathrm{sample}}/{\mathrm{A}}_{{{\mathrm{517}}}} } \right)\times 100$$

Data for total sugars, carotenoids, phenols and DPPH are presented in a table as averages for storage dates, in addition to zero date and shelf life data.

### Decay incidence

The stored mango fruits were visually inspected on a weekly basis for decay symptoms. Fruit with any signs of microbial infection or softened area was considered as decayed; decay incidence was calculated by the following equation:

4 $${\text{Decay incidence }} = {\text{ }}\left( {{\text{number of decayed fruits }}/{\text{ total number of fruits}}} \right)\times 100$$

### Fruit shelf life

Fruit shelf life was defined as the duration (in days) during which the treated fruits remained acceptable for commercial sale after the completion of the 28-day cold storage period. The treated fruits were transferred to ambient temperature and monitored daily for the development of deterioration symptoms. Shelf life was assessed based on the number of days the fruits maintained acceptable market quality. At the end of shelf life period, total sugars, carotenoids, phenolic compounds, and DPPH radical scavenging activity were determined as previously described.

#### Determination of total silver concentration

At the end of storage period samples of coated fruits pulp were acid-digested according to the method of **Altundag and Tuzen**^38^ in acid mixture (Nitric acid 70% + hydrogen peroxide 30%) using microwave digestion system (Multi-wave PRO, Anton-Paar, Graz, Austria). The silver content was determined according to the analytical procedures reported by **Zhang et al.**^39^ and **Gasbarri et al.**^40^. The silver concentration was calculated by inductively coupled plasma-optical emission spectrometry (5100 ICP-OES, Agilent, California, USA) in atomic spectroscopy lab, National Research Center, Giza, Egypt.

### Statistical analysis

The experiment was carried out in a completely randomized design (CRD), with three replicates^41^. The experimental data were subjected to analysis of variance (ANOVA) to determine effect of coating treatments on fruit quality parameters at each sampling time during storage. The statistical analysis was performed using the R software, version 4.0.5, R Core Team, Vienna, Austria. Significant differences between treatments (*p* ≤ 0.05) were assessed by the means of multiple Duncan range tests^42^.

## Results and discussion

### Particle size and zeta potential of AgNPs-SA

**Fig. 1:**
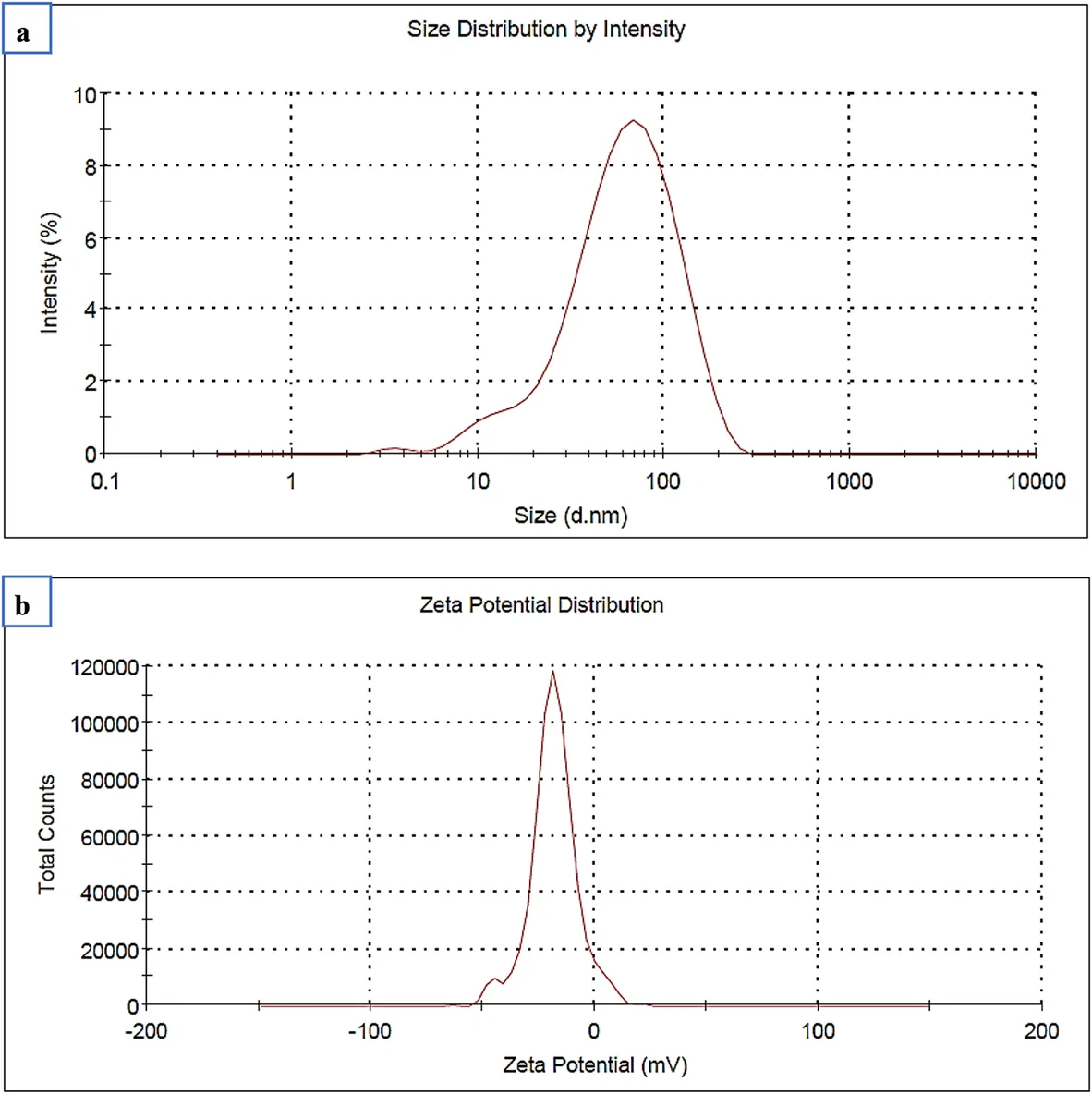
The DLS analyses of AgNPs-SA (**a**) size and (**b**) zeta potential.

Zeta potential and particle size are critical factors influencing nanoparticle stability and efficiency^43^. AgNPs-SA exhibited an average hydrodynamic diameter of 47.27 nm and a zeta potential of -20 mV (Fig. 1a, b) that reflects the potential stability of the nanomaterial, and represents a particle’s charge^44^. If every particle in suspension has a high negative or positive zeta potential, they will reject one another and will not flocculate, whereas if the particles’ zeta potential is low, there is no force to keep them from clumping and flocculating. Surface adhesion, which is related to surface charge features, has been investigated using the value of zeta potential, which is useful for understanding and forecasting interactions between particles in suspension^45^. In the current study, the zeta potential of AgNPs-SA was significantly negative (-20 mV) (Fig. 1b).

### Transmission Electron Microscopy of AgNPs-SA

**Fig. 2:**
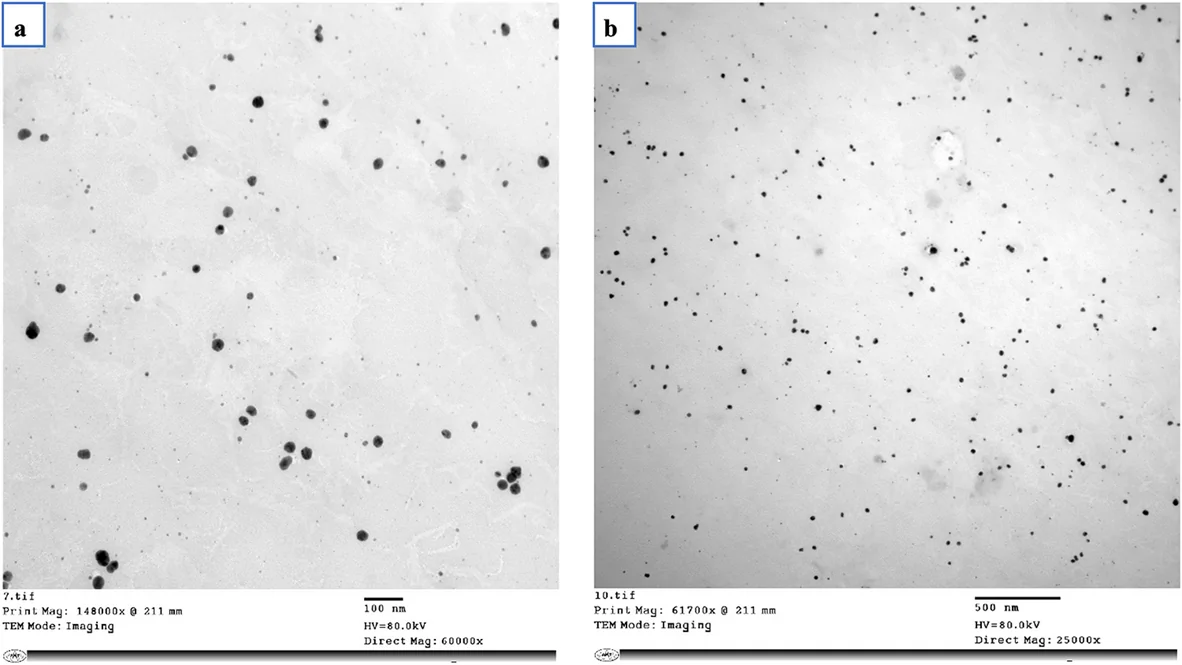
Transmission electron microscope micrographs of AgNPs-SA at different magnification levels (**a**) higher magnification image (**b**) lower magnification image.

Figure 2a and b show TEM pictures of essentially homogenous spherical shape. Based on these findings, the nanoparticles’ average size ranges between 20.9 and 33.4 nm. The photos show that the AgNPs-SA are not physically in contact with one another. A higher magnification view reveals that AgNPs form the black core of the nanoparticles, which are coated in a light gray film of salicylic acid, the organic stabilizing agent (Fig. 2a).

#### Scanning Electron Microscopy (SEM) and energy-dispersive X-ray spectroscopy (EDX) of AgNPs-SA

**Fig. 3:**
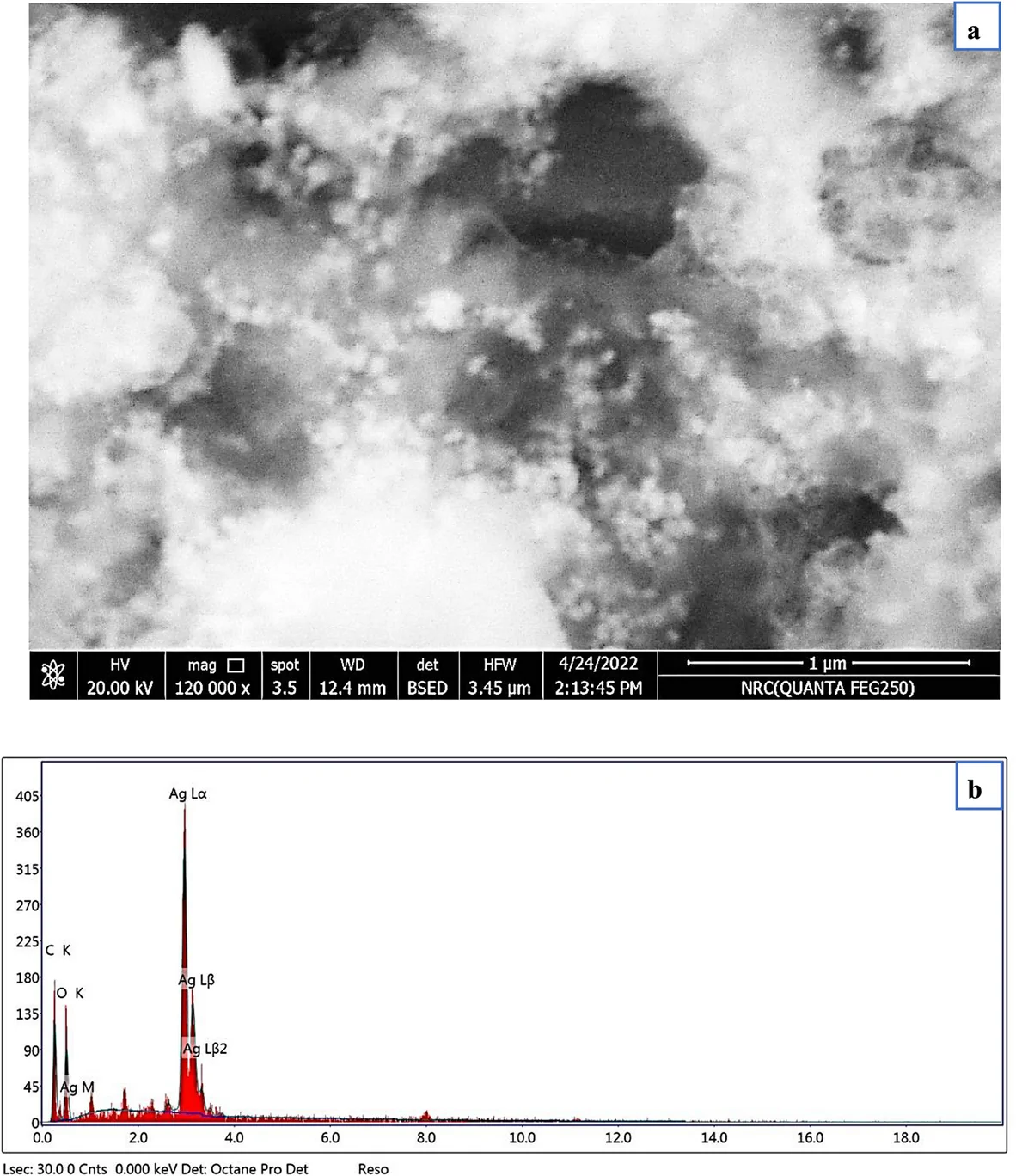
(**a**) Scanning electron microscope micrograph of AgNPs-SA, (**b**) EDX of AgNPs-SA.

Figure 3a shows the SEM picture of AgNPs-SA after laser beam treatment, and the figure depicts heavy colonization. The presence of silver nanoparticles was confirmed by EDX (energy dispersive X-ray spectroscopy), which produced peaks ranging from 0 to 4 kV (Fig. 3b). The effective synthesis of AgNPs was validated when silver nanoparticles exhibited an absorbance peak at 3 KeV of 48.69% (Table 1), indicating high silver content in the colloidal solution of silver nanoparticles. Furthermore, EDAX detected carbon and oxygen, showing that the chemical functional groups in salicylic acid stabilised AgNPs-SA^46^.

**Table 1:** Elemental analysis of EDX.

Element	Weight%	Atomic %
C K	18.64	38.37
O K	32.67	50.47
Ag L	48.69	11.16

### Fourier transform infrared

**Fig. 4:**
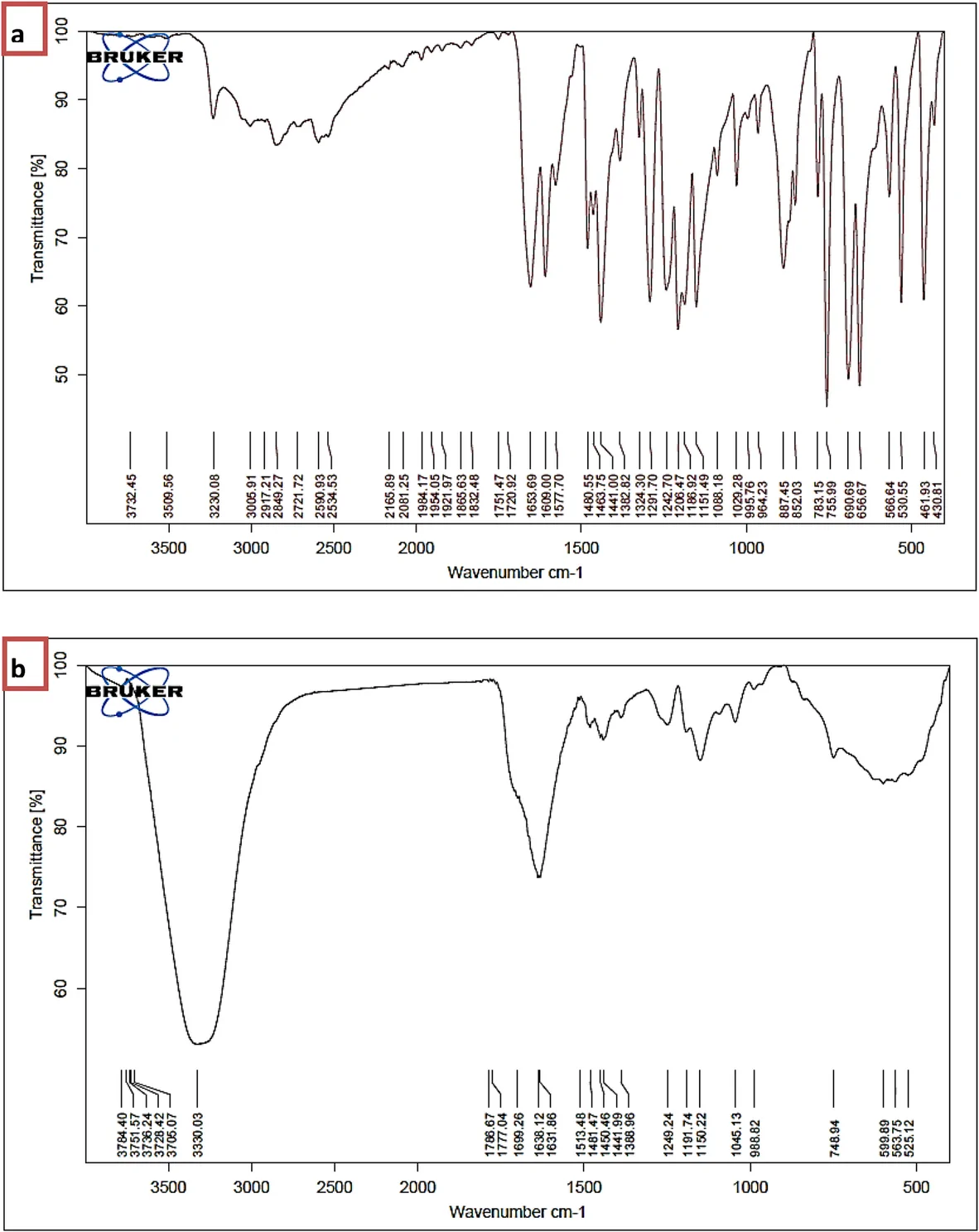
FT-IR spectrum of (**a**) salicylic acid (**b**) AgNPs-SA.

**Fig. 5:**
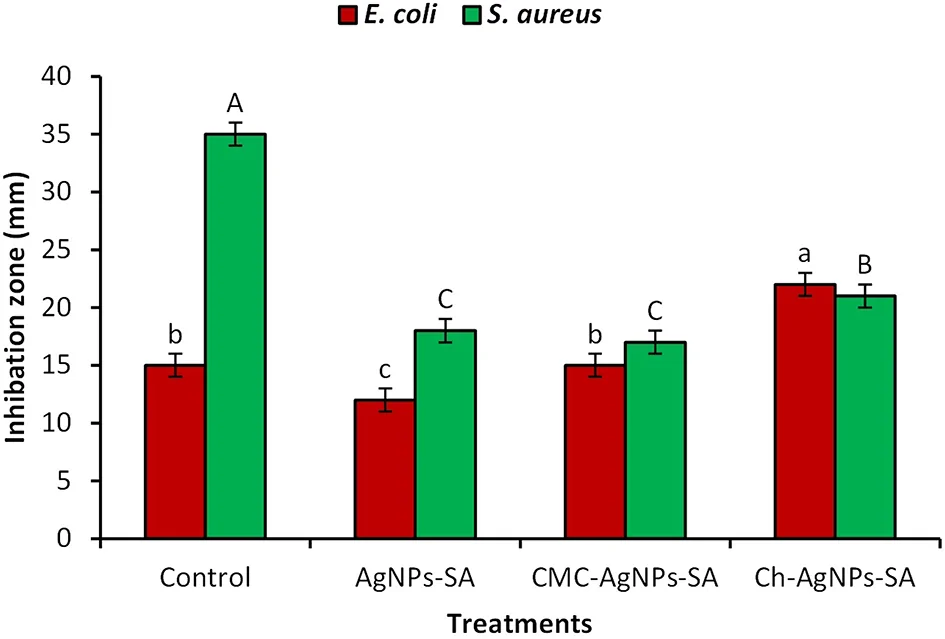
Inhibition zone diameter (mm) of the tested nano-treatments against *Escherichia coli* (red bars) and *Staphylococcus aureus* (green bars), compared to the control (polymyxin antibiotic), assessed using the well diffusion method. Vertical bars represent the standard error (SE). Bars labeled with the same letter indicate no significant difference at a 95% confidence level, as determined by Duncan’s multiple range test.

Figure 4 shows the FTIR spectra of salicylic acid (Fig. 4a) and the resulting AgNPs-SA (Fig. 5b). Prior to the reaction with AgNO_3_ solution, the FTIR spectrum of pure salicylic acid powder revealed multiple peaks showing the presence of chemical bonds and functional groups of salicylic acid (O-H, C-H, C-C, and C = O) (Fig. 4a). Figure 4b depicts the chemical changes that occur in the functional groups that are entirely responsible for the bio-reduction of AgNO_3_ by salicylic acid. The AgNPs-SA spectra exhibited peaks from 748 to 525 cm^− 1^, with absorption bands induced by vibration of chemical function groups at 3330, 1638, 1450, 1249, 1150, and 988. The AgNPs-SA spectrum verifies O-H stretch and bending with a large absorption peak at 3330 and other peaks ranging from 748 to 525 cm^− 1^. The strong absorption at 1638 cm^− 1^ is due to the carbonyl stretching vibration of carboxylate ions (-COO-), which stabilizes the silver nanoparticles and demonstrates the role of SA in the reduction and capping of Ag^+^ to Ag^46,47^.

### Antimicrobial activities of the prepared nanoparticles (NPs)

The antibacterial evaluation indicated distinct differences in activity for the treatments tested against *E coli* and *S aureus*. The maximum inhibition zone value for the control treatment against *S. aureus* was at 35 mm, while for *E. coli*, it was considerably lower at 15 mm (Fig. 5). This was in agreement with a general trend, where Gram-positive bacteria are more susceptible to antimicrobial agents due to their simpler cell wall structure, as opposed to Gram-negative bacteria, whose outer membrane provides an additional barrier to penetration of antimicrobial compound(s)^48^. Meanwhile, for both *E. coli* and *S. aureus*, the AgNPs-SA treatment showed smaller inhibition zones than that of the control, which were 12 mm and 18 mm, respectively (Fig. 5). Normally, the action of silver nanoparticles against microorganisms is due to the release of Ag⁺ ions, disruption of the cell membrane, and the production of reactive oxygen species (ROS)^49,50^. The inhibition zones achieved with CMC-AgNPs-SA were 15 and 17 mm against *E. coli* and *S. aureus*, respectively (Fig. 5), thus indicating moderate improvement. Such an improvement is probably related to the dispersion capability and stability of the nannomaterials provided by carboxymethyl cellulose (CMC), preventing their aggregation, and favoring their interaction with bacterial cells, while enhancing Ag⁺ release and ROS-mediated injuries^51^. For Ch-AgNPs-SA, the largest zones of inhibitions of 22 and 21 mm against *E. coli* and *S. aureus*, respectively, were recorded (Fig. 5). This increase is significant because of the synergistic action between chitosan, silver nanoparticles, and salicylic acid. Chitosan itself is an effective, natural antimicrobial due to its charge interaction with negatively charged bacterial plasma membranes, which increases permeability, and allows deeper penetration of the nanoparticles into the bacterial cells^48^. Another function of chitosan is to enhance the stability of nanoparticles, and promote the sustained release of the Ag⁺ ions, thereby increasing ROS formation, and inhibition of enzymes inside the bacterial cells^52^. This similar activity against both the types of bacteria indicated that chitosan effectively enhances AgNP interactions with even the Gram-negative outer membrane. In general, these results emphasize the substantial contribution of the nanomaterial toward the antibacterial performance of the material. The chitosan coated silver nanoparticles were most potent due to the combination of physicochemical stabilization and intrinsic antimicrobial activities of the biopolymer, and hence, these proved to be a promising antimicrobial nanocomposite.

### Quality assessment of mango fruits

**Fig. 6:**
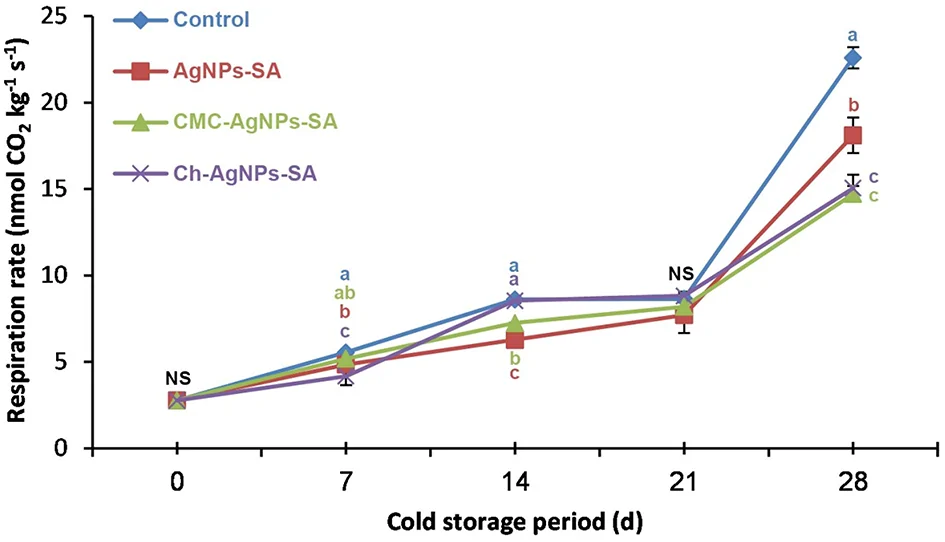
Influence of tested nano-treatments on the respiration rate of ‘Fajri Klan’ mangoes during cold storage. Each treatment is represented by a different color, with significant letters matching the corresponding treatment color for clarity. Treatments sharing the same letter on a given sampling date do not exhibit statistically significant differences at a 95% confidence level, as determined by Duncan’s multiple range test. The vertical bars represent the standard error (SE).

The data under consideration from this point forward pertains to fruit attributes and quality assessment throughout the storage period, which lasted approximately 28 days under cold storage conditions. This was followed by a variable number of days, depending on the applied treatments, representing the fruit’s shelf life. As observed in Fig. 6, the respiration rate was initially uniform at the start of the experiment (day 0) with no significant differences, however, a gradual increase was noted across all the treatments as the duration of cold storage at 13 °C progressed. This increase was particularly pronounced during the final week of the study. Additionally, it was observed that the untreated fruits exhibited the highest respiration rate compared to the other treatments throughout each week of the experiment. Upon comparing the treatments at the final sampling date (after 28 days of cold storage), it was evident that the untreated fruits exhibited the highest significant (*p* ≤ 0.05) respiration rate (22.59 nmol CO_2_ kg^− 1^ s^− 1^), followed by the fruits treated with the AgNPs-SA (18.11 nmol CO_2_ kg^− 1^ s^− 1^). Meanwhile, the treatments CMC-AgNPs and Ch-AgNPs recorded the lowest respiration rates of 14.72 and 15.05 nmol CO_2_ kg^− 1^ s^− 1^, respectively, after 28 days of cold storage.

The ripening process of climacteric fruits, such as mango, is marked by a sharp increase in respiration rate, coupled with profound metabolic transformations^53^. Coating materials function as semi-permeable barriers, regulating gas exchange and limiting the movement of solvents and moisture, thereby slowing the respiration rate, oxidative reactions, and weight loss^20,54^. Consequently, uncoated fruits exhibited a more rapid deterioration compared to their coated counterparts. The incorporation of silver nanoparticles (AgNPs-SA) significantly enhanced the water vapor barrier and mechanical strength of edible coatings^55,56^. The CMC based composite coating enriched with AgNPs-SA demonstrated minimal rise in respiration rate in mango fruits during storage^23^. This suppression of respiration effectively delayed ripening and preserved the fruit quality for a longer duration compared to the uncoated fruits. Silva et al. (2017) also observed that the respiration rate of mango fruits increased in correlation with a higher incidence of decay^57^. Chitosan coatings effectively suppressed the respiration rate in nectarine and banana fruits thereby delaying senescence and prolonging shelf life^58,59^. Additionally, these coatings strengthened the antioxidant defense system, further contributing to the reduction in respiration rate.

**Fig. 7:**
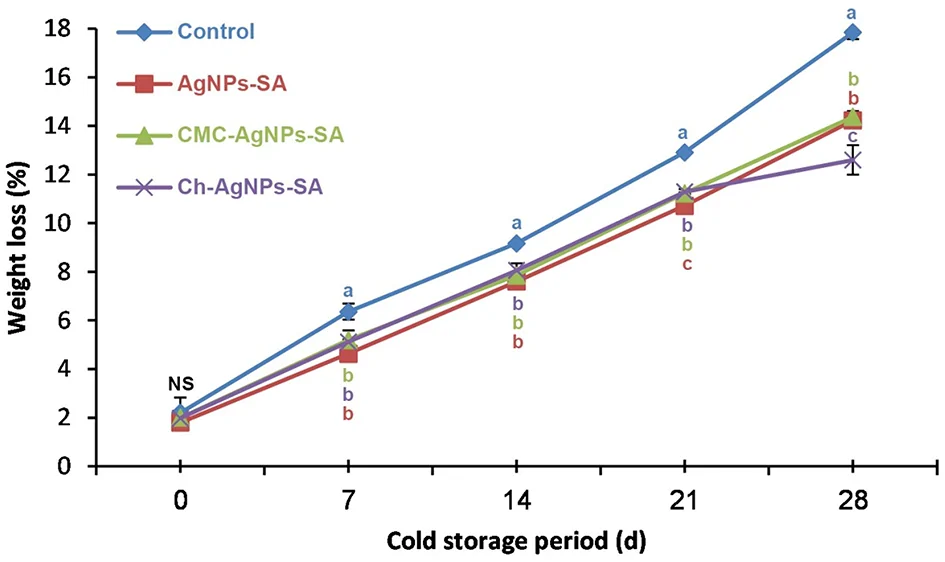
Influence of tested nano-treatments on the weight loss of ‘Fajri Klan’ mangoes during cold storage. Each treatment is represented by a different color, with significant letters matching the corresponding treatment color for clarity. Treatments sharing the same letter on a given sampling date do not exhibit statistically significant differences at a 95% confidence level, as determined by Duncan’s multiple range test. The vertical bars represent the standard error (SE).

A similar trend was also observed in weight loss in this study (Fig. 7), where ‘Fajri Klan’ mango fruits exhibited a noticeable gradual increase in weight loss compared to the other treatments during the entire period of cold storage at 13° C. This increase was significantly higher at all weekly sampling points, except for the initial date, where no significant differences were detected among the treatments. At the final sampling point, the Ch-AgNPs-SA treatment exhibited the lowest significant (*p* ≤ 0.05) weight loss rate (12.60%), followed by the other treatments, while the uncoated control fruits recorded the highest significant (*p* ≤ 0.05) weight loss (17.85%). The rate of weight loss was considerably lower in coated fruits compared to their uncoated counterparts, highlighting the efficacy of these coatings in preserving fruit quality during cold storage. Carboxymethyl cellulose (CMC) coatings incorporated with silver nanoparticles have shown to effectively minimize weight loss in citrus^60^ and mango^23^ fruits during storage. Likewise, chitosan coatings applied on guava fruits effectively minimized weight loss, and slowed the ripening process, contributing to prolonged postharvest quality preservation^61^.

**Fig. 8:**
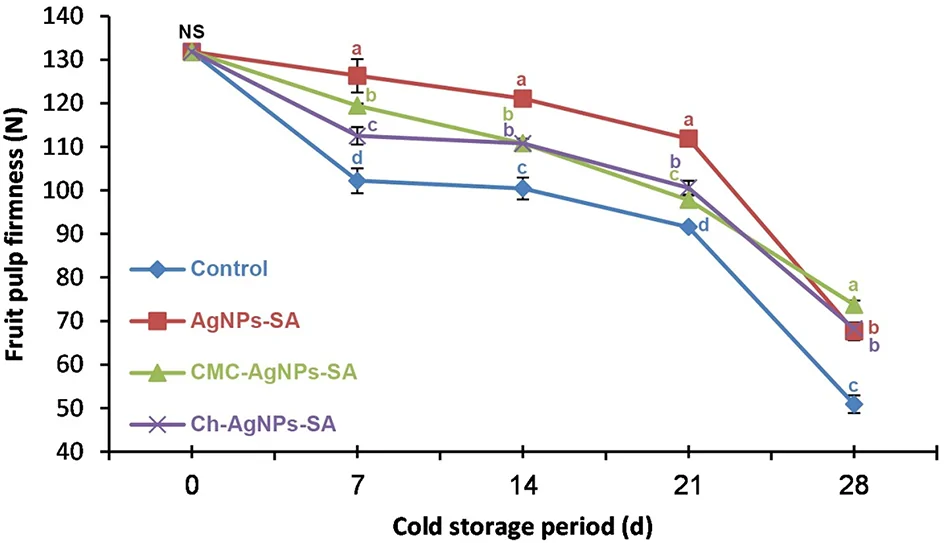
Influence of tested nano-treatments on the pulp firmness of ‘Fajri Klan’ mangoes during cold storage. Each treatment is represented by a different color, with significant letters matching the corresponding treatment color for clarity. Treatments sharing the same letter on a given sampling date do not exhibit statistically significant differences at a 95% confidence level, as determined by Duncan’s multiple range test. The vertical bars represent the standard error (SE).

As illustrated in Fig. 8, mango fruit firmness gradually declined over the course of cold storage, with the highest values recorded during the first week and the lowest values observed in the final week, after 28 days of cold storage. The CMC-AgNPs-SA treatment exhibited the highest significant (*p* ≤ 0.05) firmness value after 28 days (73.73 N), followed by the AgNPs-SA and Ch-AgNPs-SA treatments (67.65 and 68.27 N, respectively) without any significant differences observed between them. In contrast, the uncoated fruits recorded the lowest significant (*p* ≤ 0.05) firmness value (50.90 N). CMC coatings applied to mandarins and mango fruits effectively preserved fruit firmness and minimized weight loss^23,62^. Additionally, chitosan treatment delayed the climacteric peak, and maintained firmness by modulating mitochondrial respiration and inhibiting starch degradation^57^. Once mango fruits detached from the tree, the fruits undergo a series of metabolic processes, including transpiration and respiration. A strong positive correlation has been observed between weight loss and respiration rate, whereas pulp firmness exhibits a significant negative correlation with these factors^23^. In more details, the loss of fruit firmness is primarily attributed to a reduction in cellular turgidity and enzymatic degradation of the middle lamella, which involves the hydrolysis of polysaccharides within fruit cells during ripening^63,64^. Key structural polysaccharides, including starch, pectin, and hemicelluloses, progressively diminish as ripening advances^63^. The preservation of pulp firmness in coated fruits can be linked to the suppression of respiration and other ripening-associated processes during storage. This effect may be attributed to the coating’s ability to limit gas exchange, thereby reducing enzymatic activity and delaying textural softening. Moreover, the nano-coating material effectively decelerated metabolic and enzymatic activities, resulting in a slower degradation of pulp tissues^57^.

**Fig. 9:**
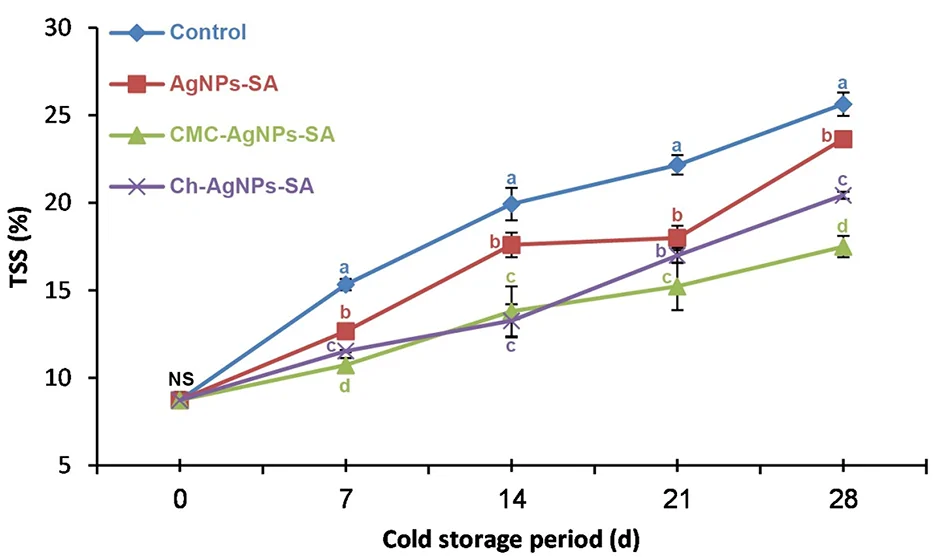
Influence of tested nano-treatments on the total soluble solids (TSS) of ‘Fajri Klan’ mangoes during cold storage. Each treatment is represented by a different color, with significant letters matching the corresponding treatment color for clarity. Treatments sharing the same letter on a given sampling date do not exhibit statistically significant differences at a 95% confidence level, as determined by Duncan’s multiple range test. The vertical bars represent the standard error (SE).

**Fig. 10:**
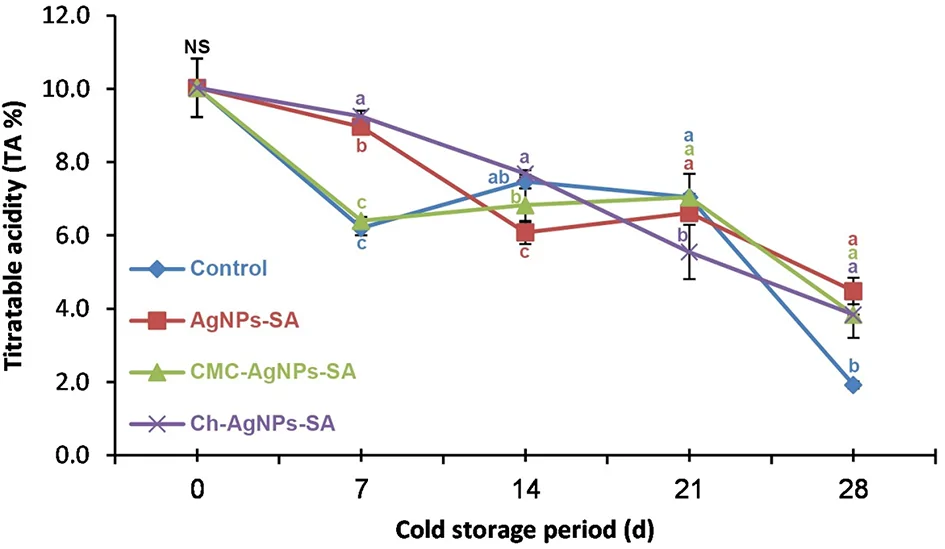
Influence of tested nano-treatments on the titratable acidity (TA) of ‘Fajri Klan’ mangoes during cold storage. Each treatment is represented by a different color, with significant letters matching the corresponding treatment color for clarity. Treatments sharing the same letter on a given sampling date do not exhibit statistically significant differences at a 95% confidence level, as determined by Duncan’s multiple range test. The vertical bars represent the standard error (SE).

**Fig. 11:**
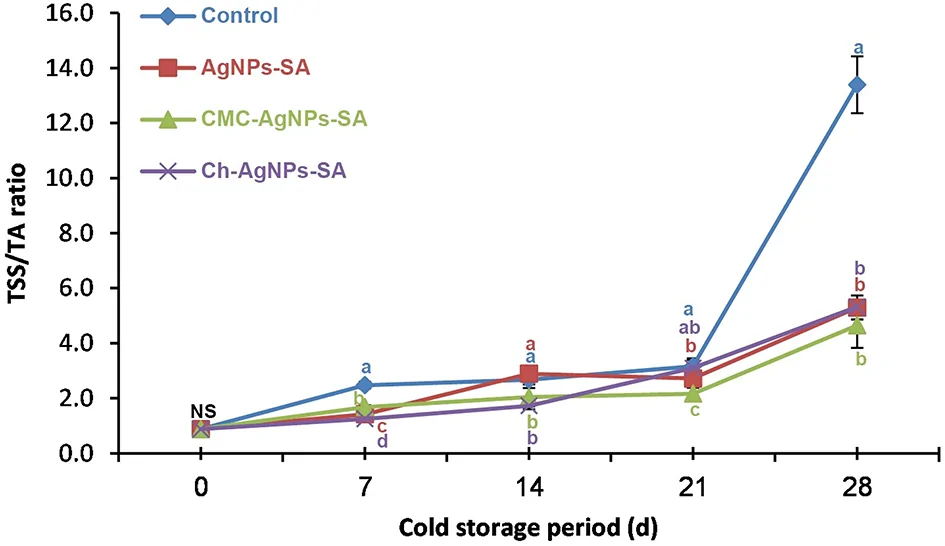
Influence of tested nano-treatments on the TSS/TA ratio of ‘Fajri Klan’ mangoes during cold storage. Each treatment is represented by a different color, with significant letters matching the corresponding treatment color for clarity. Treatments sharing the same letter on a given sampling date do not exhibit statistically significant differences at a 95% confidence level, as determined by Duncan’s multiple range test. The vertical bars represent the standard error (SE).

As observed in Fig. 9, the total soluble solids (TSS) content in the fruits gradually increased with the extension of cold storage duration. The control treatment consistently exhibited the highest TSS percentage throughout all weeks of the experiment. For instance, when comparing the first and final weeks in the control and AgNPs-SA treatments, the increase reached nearly threefold. Moreover, a slower increase was observed in both the CMC-AgNPs-SA and Ch-AgNPs-SA treatments. The control treatment recorded the highest TSS percentage, which changed from 8.73% in the first week to 25.63% in the last week of cold storage, while the CMC-AgNPs-SA and Ch-AgNPs-SA treatments exhibited the same control value of the first week (8.73%) and the lowest values during the last week (17.50 and 20.43%, respectively). The integration of silver nanoparticles (AgNPs-SA) into carboxymethyl cellulose (CMC) and chitosan coatings has been shown to effectively slow the rise in TSS in fruits such as mango^23^, pomegranate^65^, and tangerine^66^, thereby preserving their quality during storage. At first, titratable acidity (TA) was high in both treated and untreated fruits, but it gradually began to decline over the course of cold storage (Fig. 10). A steady decrease was observed with the Ch-AgNPs-SA treatment (10.03, 9.25, 7.68, 5.55, and 3.84% at 0, 7, 14, 21, and 28 days, respectively), whereas the decline was irregular in the other treatments. By the final week, after 28 days of cold storage, the control group exhibited the lowest statistically significant (*p* ≤ 0.05) acidity value (1.92%) compared to the other treatments. However, no significant differences were found among the remaining treatments, all of which showed lower acidity levels than the untreated fruits (control). In essence, as previously noted, the application of coatings slows respiration and metabolic processes, thereby reducing the excessive utilization of organic acids in respiration reactions^12^. Compared to uncoated fruit samples, edible coatings have been shown effective suppression of respiration rate, consequently limiting the depletion of titratable acids^66^. Figure 11 indicates the TSS/TA ratio, which gradually increased during the cold storage of the treated mango fruits. By the end of the 28 days of cold storage, the control recorded a ratio of 13.39, which was significantly higher (*p* ≤ 0.05) compared to that in the other nano-based treatments (AgNPs-SA, CMC-AgNPs-SA, and Ch-AgNPs-SA). The TSS/TA ratio is a key indicator of fruit quality, as it reflects the balance between sweetness and acidity. The sweet taste develops through the hydrolysis of polysaccharides, primarily starch, accompanied by a reduction in acidity and the accumulation of sugars, ultimately leading to an optimal sugar-to-acid ratio^63^.

Data in Table 2 presents measurements of total sugars, carotenoids, phenols, and antioxidant activity, highlighting the initial sampling stage, the average storage period, and shelf life. Regarding total sugars, a significant increase (*p* ≤ 0.05) was observed in the control fruits during cold storage compared to the coated fruits, which recorded notably lower values. During the 28 days of storage, the fruits exhibited a similar trend, except for those treated with Ch-AgNPs-SA, which showed significantly lower total sugar values. Total carotenoids were initially low in the fruits at the beginning of storage at 1.10 µg g^− 1^ fresh weight (FW). After the storage period, average total carotenoid values increased across all treatment groups, with the control group showing the highest increase to 2.25 µg g^− 1^ FW. During shelf life, total carotenoids continued to increase in the control and CMC-AgNPs groups, remained stable in the AgNPs group, and decreased in the Chit-AgNPs group. The untreated fruits exhibited the highest carotenoid content compared to the other treatments throughout both cold storage and shelf life. On the other hand, the total phenolic content of the fruits on day zero was 182.81 mg g^− 1^ (FW) across all treatments. This content gradually declined during the cold storage period and continued to decrease throughout the shelf-life period in all treatments, as shown in Table 2, The DPPH percentages followed a similar pattern, starting at high levels across all treatments at the beginning of storage and gradually declining throughout both the storage period and shelf-life.

**Table 2:** Influence of tested nano-treatments on the total sugars, total carotenoids, total phenol, and DPPH of ‘Fajri Klan’ mangoes during cold storage and shelf life compared to their initial levels at day-zero.

	Day-zero	Average of Storage period	Shelf life
	Total Sugar (mg g^− 1^ FW)		
Control	104.49	170.45 ^A^	194.35 ^A^
AgNPs	104.49	164.20 ^B^	172.10 ^B^
CMC-AgNPs	104.49	151.16 ^C^	170.15 ^B^
Chit-AgNPs	104.49	165.42 ^B^	152.25 ^C^
	Total Carotenoids (µg g^− 1^ FW)		
Control	1.10	2.25 ^A^	2.80 ^A^
AgNPs	1.10	2.03 ^BC^	2.25 ^C^
CMC-AgNPs	1.10	2.15 ^AB^	2.43 ^B^
Chit-AgNPs	1.10	1.95 ^C^	2.05 ^D^
	Total Phenol (mg g^− 1^ FW)		
Control	182.81	141.20 ^C^	79.035 ^A^
AgNPs	182.81	145.83 ^BC^	103.26 ^A^
CMC-AgNPs	182.81	149.51 ^B^	101.04 ^A^
Chit-AgNPs	182.81	164.69 ^A^	112.17 ^A^
	DPPH Radical Scavenging Activity (%)		
Control	339.92	249.99 ^C^	121.22 ^C^
AgNPs	339.92	289.98 ^A^	123.44 ^C^
CMC-AgNPs	339.92	269.25 ^B^	153.29 ^B^
Chit-AgNPs	339.92	268.32 ^B^	187.02 ^A^

Treatments sharing the same letter on a given sampling period do not exhibit statistically significant differences at a 95% confidence level, as determined by Duncan’s multiple range test. (FW = Fresh Weight)

**Fig. 12:**
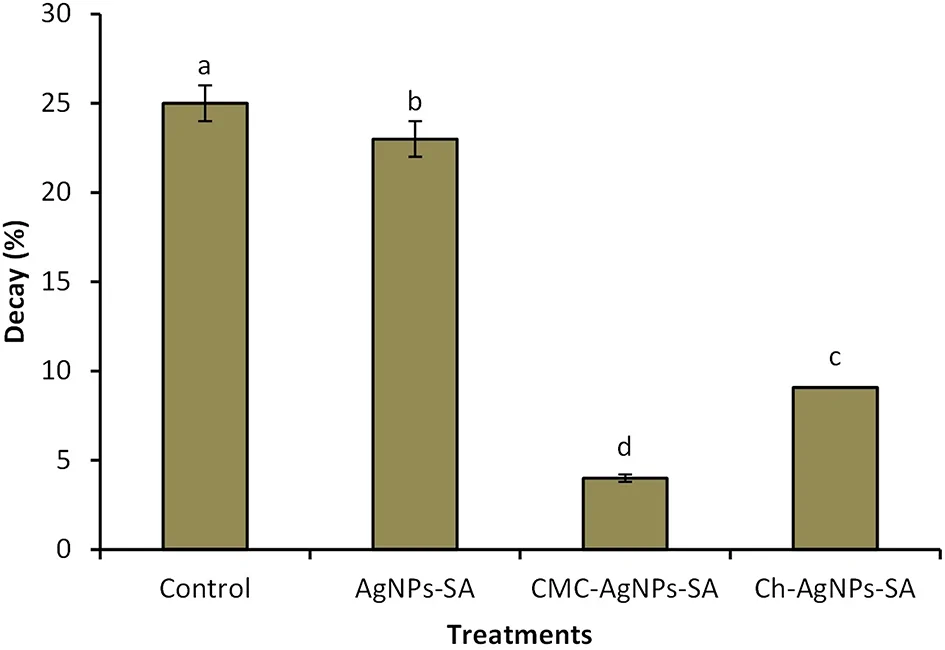
Influence of tested nano-treatments on the decay (%) of ‘Fajri Klan’ mangoes during cold storage. Treatments sharing the same letter do not exhibit statistically significant differences at a 95% confidence level, as determined by Duncan’s multiple range test. The vertical bars represent the standard error (SE).

As previously discussed, sugar accumulation increases as fruit ripens due to the hydrolysis of polysaccharides, resulting in the formation of soluble sugars during storage^63,67^. In ripe fruits, these soluble sugars primarily consist of glucose, fructose, and sucrose^68^. The application of coating effectively inhibits the conversion of complex carbohydrates into simple sugars^23^. The rapid fluctuation in total sugar content observed in the control treatment can be attributed to the respiratory burst in mango fruits, a process characterized by substantial biochemical changes that accelerate ripening^69^. The delayed ripening and slowed internal color development in coated fruits can be attributed to the reduced respiration rate, which inhibits chlorophyll degradation and/or carotenoid biosynthesis^70^. Polysaccharide-based composite coatings exhibit synergistic effects on color retention by postponing the synthesis of coloring pigments in mango fruits^20,71^. Coatings containing CMC and chitosan have been shown to preserve higher levels of total phenolics in fruits, which is essential due to their significant contribution to antioxidant properties and overall health benefits^72^. Additionally, the incorporation of CMC and chitosan in coatings helps maintain antioxidant activity during storage, a function closely associated with the presence of phenolic compounds^72^. Previous studies have reported a slower degradation rate of phenolic compounds in coated mango fruits^73^. These compounds play a crucial role in determining fruit quality attributes such as taste, flavor, and aroma, while also scavenging reactive oxygen species generated during the ripening process^74^. CMC coatings enhance the antioxidant properties of fruits by preserving phenolic content and boosting total antioxidant capacity, including DPPH radical scavenging activity. These coatings have also been shown to slow the degradation of vitamin C and other antioxidants, effectively maintaining fruit quality throughout storage^75^. Also, Chitosan-based coatings have been shown to enhance the antioxidant capacity of fruits by preserving higher levels of phenolics, anthocyanins, and flavonoids, thereby increasing DPPH radical scavenging activity. This effect is mediated through the regulation of antioxidant enzyme activities, such as catalase and peroxidase, which play a crucial role in mitigating oxidative stress within fruit cells^76,77^ (Fig. 12).

**Fig. 13:**
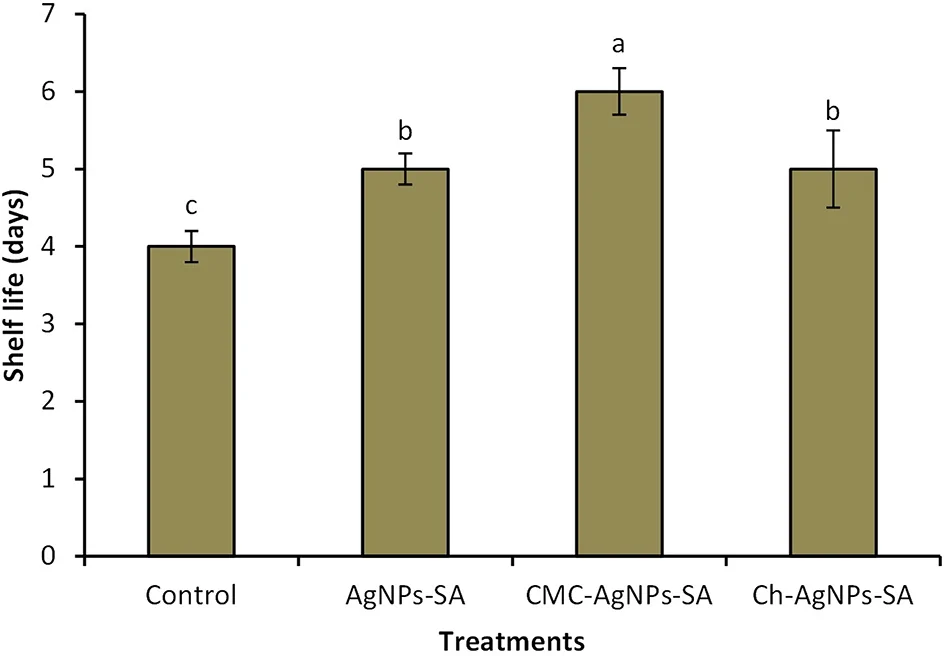
Influence of tested nano-treatments on the shelf life (days) of ‘Fajri Klan’ mangoes during cold storage. Treatments sharing the same letter do not exhibit statistically significant differences at a 95% confidence level, as determined by Duncan’s multiple range test. The vertical bars represent the standard error (SE).

Figure 13 illustrates the extent of fruit decay at the end of the experiment, where the untreated fruits (control) exhibited the highest significant decay percentage, estimated at 25%. This was followed by AgNPs-SA treatment with a value equal to 23%, and Ch-AgNPs-SA treatment (9%), while CMC-AgNPs-SA treatment recorded the lowest significant fruit decay with a value equal to 4%. The reduction in decay incidence is likely attributed to the coating’s ability to delay senescence, thereby minimizing susceptibility to pathogenic infections^20,78^.

Regarding shelf life, measured in days after the 28-day cold storage period, the treatments under study exhibited varying and statistically significant differences (*p* ≤ 0.05). The shelf life ranged from 4 days for the untreated control fruits to 5 days for those treated with AgNPs-SA and Ch-AgNPs-SA, while the CMC-AgNPs-SA-treated fruits maintained their quality for up to 6 days (Fig. 13; Table 2). CMC- and chitosan-based silver nanoparticle (AgNP) coatings have demonstrated effectiveness in delaying ripening and preserving the quality of apricot^79^, avocado^80^, citrus^81^, and mango^23,82^ fruits. By minimizing weight loss, respiration rate, and the degradation of firmness and acidity during cold storage, these coatings significantly extend the fruits’ shelf life. Moreover, the analysis for silver residues in the treated fruits yielded negative results (data not shown), confirming the absence of detectable residues across all treatments. This indicates the safety of these treatments for this application.

## Conclusions

The developed coating treatments examined in this study proved effective in enhancing key quality attributes of ‘Fajri Klan’ mangoes. They significantly minimized weight loss, slowed respiration rates, preserved fruit firmness, and reduced microbial spoilage. Characterization confirmed the formation of stable spherical silver nanoparticles with an average size of 20.9 to 33.4 nm, and negative zeta potential indicated good colloidal stability, whereas spectroscopic and elemental analyses verified the role of salicylic acid in nanoparticle reduction and stabilization. Moreover, the nanocomposite coatings demonstrated notable antimicrobial activity, with chitosan-based formulations exhibiting the strongest inhibitory effects against both Gram positive and Gram-negative bacteria, highlighting a synergistic interaction between chitosan, silver nanoparticles, and salicylic acid. Additionally, the rise in total soluble solids, sugars, and carotenoids was better regulated in the coated mangoes compared to the uncoated ones. Biochemical analysis further revealed higher retention of phenolic compounds and antioxidant activity, particularly in chitosan-based coatings, indicating enhanced protection against oxidative degradation. Fruit decay was markedly reduced in all coated treatments, with CMC based coatings yielding the lowest decay percentages, so that shelf life extended from 4 days in untreated fruits to up to 6 days in coated fruits after cold storage. Notably, no detectable silver residues were found in treated fruits, supporting the safety of the applied coatings. These findings highlight the potential of AgNP-loaded coatings, specifically CMC-AgNPs-SA and Ch-AgNPs-SA, in delaying ripening and extending the postharvest lifespan of mangoes. Collectively, the results demonstrate that these coatings offer practical benefits for improving storage conditions, prolonging marketability, and potentially boosting mango exports by reducing postharvest losses and enhancing fruit quality.

## Data Availability

Data are available upon request.
